# Blood Pressure and Haematological Indices in Twelve Communities in Ashanti, Ghana

**DOI:** 10.1155/2018/5952021

**Published:** 2018-04-05

**Authors:** Jacob Plange-Rhule, Sally M. Kerry, John B. Eastwood, Frank B. Micah, Sampson Antwi, Francesco P. Cappuccio

**Affiliations:** ^1^Division of Clinical Sciences, Renal Medicine, St George's, University of London, Cranmer Terrace, London SW17 0RE, UK; ^2^School of Medical Sciences, Kwame Nkrumah University of Science and Technology, Kumasi, Ghana; ^3^Blizard Institute, Barts and The London School of Medicine and Dentistry, Queen Mary University of London, 58 Turner Street, London E1 2AB, UK; ^4^Department of Medicine, Komfo Anokye Teaching Hospital, P.O. Box 1934, Kumasi, Ghana; ^5^Department of Paediatrics, Komfo Anokye Teaching Hospital, P.O. Box 1934, Kumasi, Ghana; ^6^Division of Health Sciences, Warwick Medical School, University of Warwick, Gibbet Hill Road, Coventry CV4 &AL, UK

## Abstract

Hypertension is the most important risk factor for cardiovascular mortality and morbidity in Sub-Saharan Africa. In western populations, high haemoglobin levels are associated with raised BP unlike in Sub-Saharan Africa where there is a paucity of data. Our study examines the association between haematological indices with BP variables. Weight, height, BP, and whole blood indices of viscosity (Hb, haematocrit, RBC count, and MCV) were measured in 921 adults (340 men, 581 women; aged 40–75) in 12 communities in Ghana. Mean values for Hb (12.3 g/dl ± 1.7 SD), haematocrit (36.7% ± 5.2), RBC (4.10 million/*μ*L ± 0.64), and MCV were lower than reference values used in Sub-Saharan Africa. Mean BMI was 21.1 ± 4.1 indicating a lean population. Systolic BP increased by 1.0 mmHg (95% CI 0.5–1.5), *p* < 0.001, for women and 0.5 (0.1–1.0), *p* = 0.027, for men per unit increase in haematocrit. Similar relationships were found for Hb and RBC but not for MCV or platelets. The relationships were weaker when adjusted for BMI, 0.7 mmHg (0.2–1.2) in women and 0.5 (0.0–1.0) in men. Findings for diastolic BP were similar. Overall haematological indices were low. We have found a significant, positive relationship between BP, Hb, Haematocrit, and RBC count in our population.

## 1. Introduction

Cardiovascular disease has become an important cause of premature death and disability in Sub-Saharan Africa [1, 2]. Hypertension is known to be the major contributor [3] and is itself a consequence of increasing urbanisation and an increasingly western lifestyle, exemplified by increasing obesity, higher salt intake, and a sedentary lifestyle.

Another contributory factor to hypertension may be blood viscosity. Plasma and whole blood viscosity have been suggested as important determinants of arterial blood pressure [4–9] both in normotensive individuals and in those with untreated hypertension. Others, meanwhile, have suggested that both blood pressure and viscosity are themselves secondary to factors such as obesity, mental stress, smoking, and physical inactivity [10]. Nonetheless, it is possible that whole blood viscosity plays a part in the genesis of stroke and coronary artery disease [11–15] and cardiac hypertrophy [16]. Of the factors contributing to whole blood viscosity, haematocrit, red cell aggregability, red cell rigidity, and plasma viscosity, haematocrit is thought to be the most important, being responsible for at least 70% of the contribution [17]. However, others have not found a correlation between blood pressure and blood viscosity in normotensives [18, 19].

Most of the evidence relating blood pressure to blood viscosity is derived from studies from the developed world. There is very little data on the relationship between blood pressure and blood viscosity from Sub-Saharan Africa [20]. In Sub-Saharan Africa, individuals have lower haemoglobin, RBC count and haematocrit [21, 22] as a result of chronic infection, malaria, and malnutrition so blood viscosity is likely to be reduced. The question is whether blood viscosity, even within the lower ranges of haemoglobin found in Sub-Saharan Africa, exhibits any relationship to blood pressure.

The study described here examines the association of haematological indices (haemoglobin, haematocrit, mean cell volume, and red cell count) with BP variables in a population-based sample of Ghanaians, with a view to determine any relation between blood viscosity and systolic and diastolic blood pressure and to explore whether any such relationships are explained by differences in body mass index (BMI).

## 2. Methods

### 2.1. Population

Adults aged 40–75 years from 12 communities (6 semiurban, 6 rural) in the Ejisu-Juaben and Kumasi Districts of the Ashanti region of Ghana took part in the study.

In brief, there were 16,965 individuals [6,597 rural, 10,368 semiurban] living in 1,460 households [750 in rural and 710 in semiurban communities]. The 12 communities had an average population [all ages] of 1,414 per community [range 562–1,966]. The adult population [≥16 years of age] was 38% in rural and 33% in semiurban communities. The population structure of these communities was comparable to that reported for Ghana as a whole. There were 2,743 adults aged 40–75 years [23]. A stratified random sample of 1,896 inhabitants aged 40–75 years was invited to take part in the study. Stratification was by age and sex within community so that the total sample selected matched the overall population structure, and there were no differences between the communities in the age and sex distribution. The proportion of the invited community population varied according to community size, with a higher proportion in smaller communities [24].

The original study referred to above [24] was intended to determine prevalence, participant awareness, treatment, and control of blood pressure and to study the effect of dietary salt reduction advice on urinary sodium and blood pressure (Control and Intervention Communities) [25].

Since this was an intervention study we chose the lower end of the age-range to be 40 years of age. This was to minimise any risk of pregnancy and its complications in participants in the “reduced salt intake” (Intervention) communities. The upper end of the age-range of 75 years was chosen as there was a steep fall in numbers of potential participants in the older age groups.

### 2.2. Questionnaire

The participants attended a registration centre set up in their community. After obtaining written informed consent, each participant was helped to complete a questionnaire to obtain information on demographic background and medical history. The Committee on Human Research Publication and Ethics of the School of Medical Sciences, Kwame Nkrumah University of Science and Technology, Kumasi, Ghana, and the Ethics Committee of St George's Healthcare NHS Trust, London, UK approved the study protocol.

### 2.3. Measurements

Height was measured without shoes using a wooden platform and a height rule to the nearest 0.5 cm. Weight was measured to the nearest 0.5 kg with manual Seca 761 scales (Vogel & Halke, Germany) after the participants had removed their outer garments and footwear. BMI was calculated as weight (kg) divided by the square of height (m^2^).

After participants had been in a relaxed sitting position for 5 minutes, blood pressure was measured using an automatic device (OMRON HEM705CP sphygmomanometer; Omron Matsusaka Co. Ltd, Japan). The appropriate cuff size (13 × 23 cm or 16 × 30 cm) was used. Three readings were taken one minute apart; the first was discarded, and the mean of the second and third readings was used in the analysis [25].

#### 2.3.1. Fasting Blood Samples

A fasting venous blood sample was taken from each participant. The samples were put into EDTA-containing vacutainer tubes and transported without delay to Komfo Anokye Teaching Hospital and refrigerated at 4°C until analysed for full blood count, haemoglobin (Hb), packed cell volume (PCV)/haematocrit, mean corpuscular volume (MCV), red cell count (RBC), and platelets using the Cell-Dyn 3700 Haematology Analyzer (Abbott Diagnostics) and also for fasting blood glucose. A diagnosis of diabetes was made if a single fasting blood glucose sample was >7.0 mmol/L or the participant was on treatment for diabetes.

### 2.4. Statistics

Men and women were compared using a random effects model. First the relationship between each viscosity variable and blood pressure was estimated using random effects regression in both men and women separately, and allowing for clustering by community. Age, BMI, and smoking were then added to the model. BMI was fitted as a linear and quadratic term as previous research has shown this relationship may not be linear and a quadratic term gives a better fit [26, 27]. Interactions terms were added to see if the relationship between blood pressure and viscosity was significantly different in men and women. The relationships between blood pressure and age and body mass index tend to be different in men and women in this population [26]. Interactions terms with sex were fitted for all main effect. Regression coefficients for the relationship between blood pressure and each viscosity are presented with 95% confidence intervals. Mean values of systolic and diastolic blood pressure were calculated for each quintile of haematocrit for men and women separately and displayed graphically.

Blood pressure readings were adjusted for time of day [25]. Summary statistics are given as mean (SD). *p* values less than 0.05 were considered significant.

All analyses were conducted using Stata Statistical Software (StataCorp. Stata statistical software: release 4.0. College Station, TX: StatCorp; 1995).

## 3. Results

Of the 1,896 subjects invited to participate, 1,013 took part in the study. Full haematological measurements were available in 921 (340 men, 581 women). The majority of the participants (94%) were from the Ashanti tribe. Farming and trading were the main occupations [23].

**Figure 1:**
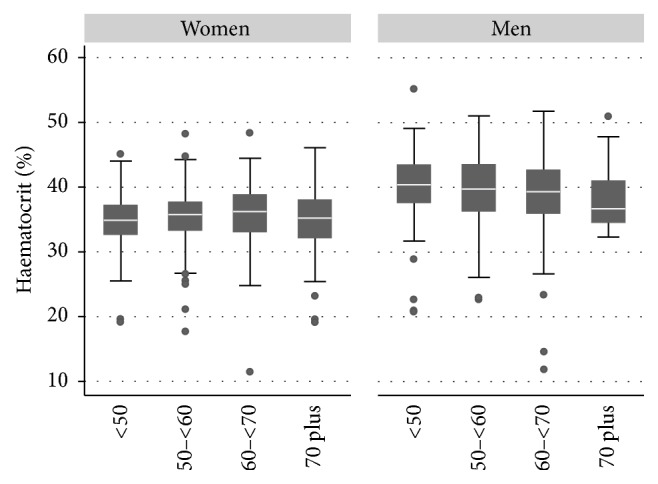
Haematocrit by age and sex; box and whisker plot showing median and interquartile range of haematocrit by age for 340 men and 581 women.

Mean values for all haematological indices except MCV were at or below the bottom of the corresponding reference range (Table 1) and some individuals had values well below the normal ranges (Figure 1). Whilst haematocrit falls with age in men, it first rises and then falls in women (Figure 1).

Overall haematocrit, haemoglobin, red blood count, and mean corpuscular volume were significantly higher in men than in women.

Mean BMI was 21.1 (4.1) kg/m^2^. BMI, though relatively low in both, was higher in the women than the men (21.6 v 20.3 kg/m^2^  *p* < 0.05); mean blood pressure was 128 (26) mmHg (systolic) and 76 (13) mmHg (diastolic); both systolic and diastolic blood pressures were similar in men and women.

Table 2 shows all the models used in our study to investigate the relationship between measures of blood viscosity and blood pressure. For women, systolic blood pressure increased by 1.0 mm Hg for each unit increase in haematocrit. (95% confidence interval 0.5 to 1.5); *p* < 0.001. After adjusting for age and body mass index, the figure fell to 0.7 mmHg per unit increase in haematocrit (95% CI 0.2 to 1.2); *p* = 0.004. Similar relationships were found for haemoglobin and red blood count with both systolic and diastolic blood pressure.

Slopes in the women were consistently steeper than in the men but none of the interactions achieved statistical significance.

There was no evidence of a relationship with blood pressure for either mean corpuscular volume or platelets (Table 2). Adjusting for age, body mass index and smoking in men did not alter these findings (data not shown).

**Figure 2:**
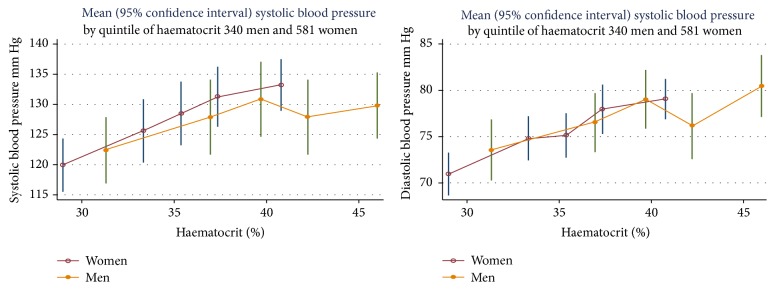
Mean blood pressure by quintile of haematocrit for men and women.

Figure 2 shows the mean blood pressure for quintiles of haematocrit. The data indicate that where men and women have the same values for haematocrit, the slopes of blood pressure are similar. However, for higher values of haematocrit, the rise in BP with a unit increase in haematocrit is less than at lower values.

The relationship between blood pressure and mean corpuscular volume or platelet count was not statistically significant for either men or women. Smoking rates among the participants were low and those that smoked only smoked an average of 4 cigarettes a day. Adjusting for smoking made a negligible difference to the relationships between blood pressure and viscosity.

## 4. Discussion

In our study, in 12 communities in the Ashanti Region of Ghana, we have described the haematological indices for healthy Ghanaians. We have shown that the subjects had low levels of haematocrit and haemoglobin, with values similar to what has been in found in populations in other countries in Sub-Saharan Africa [28, 29]. The haematological indices reported in this study are low in comparison to the frequently quoted reference ranges determined from studies in the developed world [30, 31].

In this population, in both men and women, we have shown a significant increase in both systolic and diastolic blood pressure with higher values of haematocrit, haemoglobin, and red blood count even after allowing for age and body mass index. Systolic blood pressure increased by 1.0 (0.5 to 1.5) mm Hg for each unit increase in haematocrit for women and 0.5 (0.1 to 1.0). After adjusting for age and body mass index, the figure fell to 0.7 (0.2 to 1.2) mmHg per one percent increase in haematocrit in women but remained unchanged at 0.5 (0.0 to 1.0) in men.

A number of studies have demonstrated that both measured blood viscosity and haematocrit increased with increasing blood pressure [4, 13]; and haematocrit correlated significantly with both systolic and diastolic blood pressure [13, 32]. The relationship was true both in normotensives and in untreated hypertensives. In a population study, a similar relationship was found between haematocrit and blood pressure with mean haematocrit being significantly higher in hypertensives than in normotensives for both sexes, after adjusting for age [33]. In this study untreated hypertensives and drug-treated hypertensives were found to have similar haematocrit but a significantly lower blood pressure in the treated group. This finding persisted on exclusion of hypertensives treated with diuretics from the analysis. Thus, the possible diuretic treatment-induced reductions in plasma volume did not seem to have any effect on mean haematocrit in this group.

Devereux et al. [10] found that whole blood viscosity was higher in untreated hypertensives than in normotensives. However, the authors failed to find a relationship between haematocrit and whole blood viscosity in their hypertensive subjects. One possible explanation for this in our study could be that mechanisms other than through a raised whole blood viscosity may be responsible for the positive association between blood pressure and blood haematocrit described in our study. Indeed, important factors contributing to whole blood viscosity other than haematocrit are circulating fibrinogen, red cell aggregation, and red cell deformability. Support for the effect of fibrinogen in raising blood pressure was suggested by Fowkes et al. [7], an association that was found to be independent of smoking, alcohol intake, and BMI. Salazar-Vazquez et al. [34], in a study of diabetics, found a statistically significant U-shaped/bimodal relationship between haematocrit and mean arterial blood pressure; the control subjects showed a nonsignificant increase in blood pressure with an increase in haematocrit. In contrast, De Simone et al. [6] have shown an inverse relationship between systolic blood pressure and whole blood viscosity and haematocrit in a homogeneous population of American Indians greater than 80% of who were either overweight or frankly obese and many of whom were Type II diabetics (43%) or smokers (30%). They demonstrated no relationship between diastolic blood pressure and haematocrit. The high percentage of Type II diabetes mellitus and smoking in the population used in the study by De Simone et al. [6] may have influenced the measures of blood viscosity and hence the relationship between blood viscosity and hypertension.

In summary, our data support those of Gori et al. [5] but do not support either a U-shaped curve [31] or an inverse relationship between blood pressure and haematocrit [6].

Our study was carried out in a group of unselected individuals from African communities. The population was unusually homogeneous (94% Ashanti tribe) and lean and only 30 of the 921 participants were taking antihypertensive drugs. We have therefore been able to observe the relationship between blood pressure and indirect measures of viscosity virtually uninfluenced by ethnic differences and drug treatment. Few participants in the study suffered from diabetes (4.0%) and smoking rates were relatively low (7%) so the possible influence of smoking and diabetes mellitus on haematocrit and the results of this study were minimised.

In our study, we used haemoglobin, haematocrit, and red blood cell count as indirect measures of blood viscosity to assess the relationship between viscosity and blood pressure and did not determine whole blood viscosity. We also did not measure plasma proteins.

The importance of our study is that it details the haematological indices of healthy Africans. As many of the values fall outside the standard reference values in common use in Ghana, there will be implications for clinical decision-making and patient care.

Currently, anaemia is common in many populations in Sub-Saharan Africa but as Africa-wide public health initiatives directed at improving health begin to take effect it is likely that the prevalence of anaemia will fall, particularly in women. If the relationship between blood pressure and haematocrit is causal then the correction of anaemia will tend to be accompanied by a population rise in blood pressure. Such an increase in blood pressure is likely to be exacerbated by the continuing migration of rural Africans to cities, with the consequent accompanying increase in body mass index. The importance of our results is that higher levels of haematological indices are associated with higher blood pressure independent of BMI.

We hope that the findings of this study will inform the WHO, Governments, and other policy-makers in formulating their plans for reducing the burden of noncommunicable diseases, especially hypertension, in Africa.

## 5. Conclusions

We have measured haematological indices in healthy Ghanaians living in rural and semiurban communities and found that the values are significantly lower than reference values originating in the developed world but in current use in most of Sub-Saharan Africa.

We have found that blood pressure rises as blood viscosity measured by indirect measures (haemoglobin, haematocrit, and red cell count) rises for both men and women.

## Figures and Tables

**Table 1:** Height, weight, BMI, and systolic and diastolic BP versus gender. Results are means (SD).

		Men	Women	All
		(*n* = 340)	(*n* = 581)	(*n* = 921)
Age	Years	54.6 (10.8)	54.9 (11.6)	54.8 (11.3)
Height	Cm	167 (7)^*∗∗∗*^	157 (7)	160 (8)
Weight	Kg	56 (10)^*∗∗∗*^	53 (12)	54 (11)
BMI	kg/m^2^	20.2 (3.0)^*∗∗∗*^	21.6 (4.6)	21.1 (4.1)
Systolic BP	mm Hg	128 (25)	128 (27)	128 (26)
Diastolic BP	mm Hg	77 (14)	76 (13)	76 (13)
Current smoker	*n* (%)	64 (19)	1 (0)	65 (7)
Haematocrit/packed cell volume	%	39.3 (5.5)^*∗∗∗*^	35.2 (4.4)	36.7 (5.2)
Haemoglobin	g/dL	13.2 (1.9)^*∗∗∗*^	11.8 (1.4)	12.3 (1.7)
Red blood count	×10^12^/L	4.33 (0.69)^*∗∗∗*^	3.96 (0.58)	4.10 (0.64)
Mean corpuscular volume	Fl	91.0 (8.5)^*∗∗*^	89.5 (7.7)	90.0 (8.0)
Platelets	×10^12^/L	160 (90)^*∗*^	175 (89)	169 (89)

Significance tests for *Men *versus* Women* given under *Men*. ^*∗*^*p* < 0.05; ^*∗∗*^*p* < 0.01; ^*∗∗∗*^*p* < 0.001.

**Table 2:** Systolic BP and diastolic BP (adjusted and unadjusted for BMI) by gender versus measures of viscosity: regression coefficients.

	Men (*n* = 340)	Women (*n* = 581)	Interaction^5^ (*n* = 921)
*Systolic blood pressure*			
Haematocrit (%)			
Haematocrit only^1^	0.54 (0.06 to 1.01)^*∗*^	1.00 (0.51 to 1.49)^*∗∗∗*^	0.47 (−0.22 to 1.16)
Adjusted for age^2^	0.67 (0.20 to 1.14)^*∗∗*^	0.91 (0.44 to 1.37)^*∗∗∗*^	0.24 (−0.42 to 0.90)
Adjusted for age, BMI^3^	0.50 (0.03 to 0.97)^*∗*^	0.69 (0.22 to 1.15)^*∗∗∗*^	0.17 (−0.50 to 0.83)
Adjusted for age, BMI, smoking^4^	0.49 (0.02 to 0.96)^*∗*^	#	#
Haemoglobin (g/dL)			
Haemoglobin only^1^	1.58 (0.16 to 3.00)^*∗*^	3.43 (1.90 to 4.95)^*∗∗∗*^	1.85 (−0.23 to 3.94)
Adjusted for age^2^	2.03 (0.63 to 3.44)^*∗∗*^	3.33 (1.90 to 4.76)^*∗∗∗*^	1.33 (−0.67 to 3.33)
Adjusted for age, BMI^3^	1.46 (0.05 to 2.87)^*∗*^	2.73 (1.27 to 4.19)^*∗∗∗*^	1.25 (−0.78 to 3.29)
Adjusted for age, BMI, smoking	1.47 (0.06 to 2.87)^*∗*^	#	#
Red blood count (×10^12^/L)			
Red blood count only^1^	4.6 (0.8 to 8.4)^*∗*^	7.3 (3.6 to 11.0)^*∗∗∗*^	2.70 (−2.67 to 8.07)
Adjusted for age^2^	5.7 (2.0 to 9.5)^*∗∗*^	7.0 (3.5 to 10.4)^*∗∗∗*^	1.27 (−3.89 to 6.43)
Adjusted for age, BMI^3^	4.3 (0.6 to 8.1)^*∗*^	5.5 (2.0 to 9.0)^*∗∗∗*^	1.20 (−4.01 to 6.37)
Adjusted for age, BMI, smoking	4.3 (0.6 to 8.1)^*∗*^	#	#
Mean corpuscular volume (fL)			
Mean corpuscular volume only	−0.04 (−0.36 to 0.27)	−0.09 (−0.38 to 0.20)	0.03 (−0.40 to 0.45)
Platelets (×10^12^/L)			
Platelets only	0.01 (−0.02 to 0.04)	−0.01 (−0.03 to 0.02)	0.02 (−0.02 to 0.06)
*Diastolic blood pressure*			
Haematocrit (%)			
Haematocrit only^1^	0.44 (0.18 to 0.71)^*∗∗∗*^	0.66 (0.42 to 0.90)^*∗∗∗*^	0.19 (−0.16 to 0.54)
Adjusted for age^2^	0.45 (0.19 to 0.72)^*∗∗∗*^	0.64 (0.40 to 0.88)^*∗∗∗*^	0.15 (−0.20 to 0.50)
Adjusted for age, BMI^3^	0.36 (0.09 to 0.62)^*∗∗*^	0.51 (0.27 to 0.75)^*∗∗∗*^	0.11 (−0.24 to 0.46)
Adjusted for age, BMI, smoking	0.35 (0.08 to 0.61)^*∗∗*^	#	#
Haemoglobin (g/dL)			
Haemoglobin only^1^	1.36 (0.58 to 2.14)^*∗∗∗*^	2.25 (1.51 to 2.99)^*∗∗∗*^	0.90 (−0.16 to 1.96)
Adjusted for age^2^	1.40 (0.61 to 2.19)^*∗∗∗*^	2.23 (1.50 to 2.96)^*∗∗∗*^	0.83 (−0.22 to 1.89)
Adjusted for age, BMI^3^	1.07 (0.27 to 1.86)^*∗∗*^	1.82 (1.07 to 2.56)^*∗∗∗*^	0.77 (−0.31 to 1.84)
Adjusted for age, BMI, smoking	1.09 (0.30 to 1.88)^*∗∗*^	#	#
Red blood count (×10^12^/L)			
Red blood count only^1^	3.50 (1.40 to 5.60)^*∗∗∗*^	4.38 (2.57 to 6.20)^*∗∗∗*^	0.85 (−1.90 to 3.60)
Adjusted for age^2^	3.61 (1.48 to 5.74)^*∗∗∗*^	4.31 (2.52 to 6.11)^*∗∗∗*^	0.65 (−2.10 to 3.39)
Adjusted for age, BMI^3^	2.83 (0.71 to 4.96)^*∗∗*^	3.33 (1.53 to 5.12)^*∗∗∗*^	0.44 (−2.31 to 3.19)
Adjusted for age, BMI, smoking	2.83 (0.71 to 4.95)^*∗∗*^	#	#
Mean corpuscular volume (fL)			
Mean corpuscular volume only	−0.03 (−0.20 to 0.14)	−0.02 (−0.16 to 0.13)	−0.00 (−0.22 to 0.22)
Platelets (×10^12^/L)			
Platelets only	0.00 (−0.01 to 0.02)	0.00 (−0.01 to 0.02)	−0.00 (−0.02 to 0.02)

^1^Model: BP = *β*_0_ + *β*_1_ × *V* where *V* is viscosity variable; ^2^Model: BP = *β*_0_ + *β*_1_ × *V* + *β*_2_  × age; ^3^Model: BP = *β*_0_ + *β*_1_ × *V* + *β*_2_  × age + *β*_3_  × BMI + *β*_4_  × BMI^2^; ^4^Model: BP = *β*_0_ + *β*_1_ × *V* + *β*_2_  × age + *β*_3_  × BMI + *β*_4_  × BMI^2^ + *β*_3_  × smoking; ^5^Model includes all interactions with sex for main effects in model. ^#^There was only one female smoker hence there is no regression coefficient given for adjusting for smoking in women; ^*∗*^*p* < 0.05; ^*∗∗*^*p* < 0.01; ^*∗∗∗*^*p* < 0.001.
